# Spotlight on Nerves: Portable Multispectral Optoacoustic Imaging of Peripheral Nerve Vascularization and Morphology

**DOI:** 10.1002/advs.202301322

**Published:** 2023-04-24

**Authors:** Dominik Jüstel, Hedwig Irl, Florian Hinterwimmer, Christoph Dehner, Walter Simson, Nassir Navab, Gerhard Schneider, Vasilis Ntziachristos

**Affiliations:** ^1^ Institute of Biological and Medical Imaging Helmholtz Zentrum München D‐85764 Neuherberg Germany; ^2^ Institute of Computational Biology Helmholtz Zentrum München D‐85764 Neuherberg Germany; ^3^ Chair of Biological Imaging at the Central Institute for Translational Cancer Research (TranslaTUM) School of Medicine Technical University of Munich D‐81675 Munich Germany; ^4^ Department of Anesthesiology and Intensive Care School of Medicine Klinikum Rechts Der Isar Technical University of Munich D‐81675 Munich Germany; ^5^ Department of Orthopaedics and Sport Orthopaedics School of Medicine Klinikum Rechts Der Isar Technical University of Munich D‐81675 Munich Germany; ^6^ Chair for Computer Aided Medical Procedures and Augmented Reality Technical University of Munich D‐80333 Munich Germany; ^7^ Munich Institute of Robotics and Machine Intelligence Technical University of Munich D‐80992 Munich Germany

**Keywords:** neuropathy, optoacoustic imaging, peripheral nerves, photoacoustic imaging, spectral unmixing

## Abstract

Various morphological and functional parameters of peripheral nerves and their vascular supply are indicative of pathological changes due to injury or disease. Based on recent improvements in optoacoustic image quality, the ability of multispectral optoacoustic tomography, to investigate the vascular environment and morphology of peripheral nerves is explored in vivo in a pilot study on healthy volunteers in tandem with ultrasound imaging (OPUS). The unique ability of optoacoustic imaging to visualize the vasa nervorum by observing intraneural vessels in healthy nerves is showcased in vivo for the first time. In addition, it is demonstrated that the label‐free spectral optoacoustic contrast of the perfused connective tissue of peripheral nerves can be linked to the endogenous contrast of hemoglobin and collagen. Metrics are introduced to analyze the composition of tissue based on its optoacoustic contrast and show that the high‐resolution spectral contrast reveals specific differences between nervous tissue and reference tissue in the nerve's surrounding. How this showcased extraction of peripheral nerve characteristics using multispectral optoacoustic and ultrasound imaging could offer new insights into the pathophysiology of nerve damage and neuropathies, for example, in the context of diabetes is discussed.

## Graphical abstract

Multispectral optoacoustic tomography in combination with ultrasound is the first clinical handheld system able to visualize the vasa nervorum and the internal structure of peripheral nerves in healthy volunteers in vivo, and additionally links the image contrast to multiple endogenous chromophores, including oxygenated and deoxygenated hemoglobin, lipids, and water.

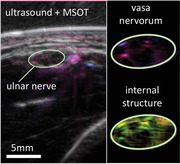

## 1 Introduction

Localization of peripheral nerves and investigation of their morphology and function is important in clinical medicine and research, for example, to identify nerves for regional anesthesia or during surgery, and for studying the mechanisms underlying peripheral neuropathies. In current clinical routine, peripheral nerves are dynamically localized with ultrasonography, based on anatomical landmarks along the nerve's progression and via the honeycomb‐like appearance of the fascicular structure of nerves.^[^ ^1^, ^2^ ^]^ Although ultrasound‐guided regional anesthesia has been shown to improve the success rate and safety of the procedure,^[^ ^2^ ^]^ electrical nerve stimulation is sometimes needed for verification due to a lack of specificity of ultrasound contrast.

US is also employed for visualizing morphological alterations associated with disease and injury,^[^ ^3^, ^4^ ^]^ in particular for diagnosing neuropathies that correlate with a change in the neural cross‐sectional area.^[^ ^4^ ^]^ However, most acquired axonal neuropathies, including diabetic neuropathy, are not associated with specific morphological changes detectable with US, e.g., a significant nerve enlargement.^[^ ^4^ ^]^ Vascularization and oxygenation are important features for clinical assessment of peripheral nerve function.^[^ ^5^ ^]^ Moreover, patients with diabetes^[^ ^6^ ^]^ and peripheral demyelinating diseases^[^ ^7^ ^]^ exhibit changes in their neural water and fat content. Existing radiological modalities have insufficient capabilities to image these parameters, in particular in relation to portable usage. The functional abilities of US imaging are limited to visualizing intraneural blood flow using the Doppler function, which is only sensitive enough to detect substantially increased blood flow in progressed stages of neuropathy.^[^ ^3^ ^]^ Other radiological methods such as magnetic resonance imaging (MRI) or positron emission tomography (PET) can identify structural damage and edema^[^ ^8^, ^9^ ^]^ or regions of high metabolic activity,^[^ ^8^ ^]^ but are expensive, not ubiquitously available,^[^ ^10^ ^]^ not suited for all patient collectives (e.g., PET—pregnant, MRI—pacemaker), and cannot assess the functionality of the vasa nervorum.^[^ ^9^ ^]^ Invasive approaches have also been considered for functional nerve investigations (e.g., endoscopic fluorescence‐lifetime (sodium fluorescein) spectrophotometry of blood oxygenation in the nerve,^[^ ^11^ ^]^ microscopic assessment of biopsies of the sural nerve, or minimally invasive skin biopsies addressing distal small fiber abnormalities^[^ ^12^ ^]^), however they are not suitable for monitoring purposes and pose a greater risk to the patient. These limitations explain why physical examinations and electrophysiology remain the standard for clinical diagnosis of peripheral neuropathy.

Optoacoustic (OA, also photoacoustic) imaging has been proposed as a noninvasive modality that complements existing peripheral nerve imaging approaches by providing optical contrast in tissue dynamically and without harmful radiation exposure. Pilot studies in animal models have demonstrated the feasibility of resolving nerve and vascular features using OA microscopy or macroscopy.^[^ ^13^, ^14^, ^15^, ^16^ ^]^ A single‐wavelength pilot study in humans also showcased the ability of OA imaging to resolve the vasculature around nerves. Nevertheless, no systematic study to our knowledge has investigated the performance of OA imaging in human nerves in vivo. Critically, no demonstration has built on the ability of multispectral optoacoustic tomography (MSOT) to identify endogenous chromophores (i.e., oxygenated and deoxygenated hemoglobin, lipids or water) in nerves by analyzing spectral optoacoustic data. MSOT has already proven its value in clinical studies by assessing the vasculature and vascular dynamics in tissues, and visualizing small blood vessels with a performance superior to clinical US.^[^ ^17^, ^18^, ^19^ ^]^ MSOT also resolves tissue oxygenation and metabolic parameters^[^ ^20^, ^21^, ^22^, ^23^, ^24^, ^25^ ^]^ without the use of contrast agents (label‐free operation) or ionizing radiation. Such capabilities would make MSOT a valuable addition to existing nerve imaging modalities by improving contrast for nerve localization and providing new biomarkers for the assessment of nerve health, for example, in the context of diabetes. However, applying MSOT to nerve imaging requires suitable data processing and analysis to overcome the detrimental effects of light absorption in superficial tissue layers and electrical noise in the imaging system.

Recent enhancements in OA image quality^[^ ^26^, ^27^, ^28^, ^29^ ^]^ allow MSOT to obtain high‐resolution spectral OA data that potentially yields more reliable spectral statistics. For example, correlations between spectral components in regions of interest (ROIs) may detect changes in the relative proportions of tissue components. Based on this development, we herein explore the performance of MSOT for imaging human peripheral nerves in vivo. We performed a pilot study in which three major distal nerves of the brachial plexus are imaged with a handheld optoacoustic‐ultrasound (OPUS) system in the upper arm of healthy volunteers. Using the spatial and spectral dimensions of MSOT, we explore its performance in revealing the vasa nervorum and the internal structure of peripheral nerves. Of particular interest was assessing MSOT's ability to observe small intraneural vessels in healthy peripheral nerves, which has previously not been possible with a handheld system in vivo.^[^ ^3^ ^]^ Visualization of physiological intraneural vasculature would provide a reference for the detection of early signs of pathological perfusion prior to loss of neural function.

We show that data‐driven unmixing methods afford a detailed picture of the vasa nervorum, including relative blood oxygenation and the internal structure of peripheral nerves by identifying various spectral components that relate to specific chromophores and their variations due to fluence attenuation in the raw spectral data. Individual fascicles can be identified via MSOT contrast of the nerve's connective tissue and its vascular supply. This level of contrast and resolution in multispectral OA images has not been demonstrated previously with a clinical hand‐held system in vivo.

In addition, we highlight the ability of MSOT's high‐resolution spectral contrast to sense differences in tissue composition. We introduce metrics to quantify tissue‐specific features related to the mixing behavior of different spectral unmixing components and demonstrate that these metrics capture the specific spectral contrast of nervous tissue and link it to blood, lipid, and collagen contrast. A refinement of the analysis that investigates clusters in the data associated with specific tissue contrasts reveals fine nuances in spectral contrast, which are potentially sensitive to pathological changes in the substructures of nerves. Our data‐driven analysis of raw spectral data complements approaches that try to correct the effects of light attenuation prior to data analysis.^[^ ^30^ ^]^ We discuss how OPUS has demonstrated the most detailed anatomical and functional visualization of nerves achieved so far and elaborate on applications that could give access to early features of neuropathy, potentially leading to earlier diagnosis and deeper insights into pathophysiology.

## 2 Results

### 2.1 OPUS Imaging of Peripheral Nerves

**Figure 1:**
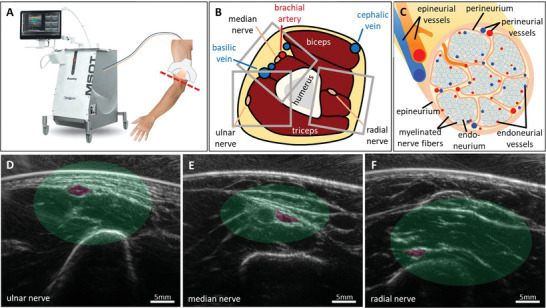
Data acquisition and anatomy. A) Co‐registered ultrasound and OA images of the three major peripheral nerves of the upper limb were acquired proximal of the elbow (red dashed line) with an iThera medical acuity echo prototype. B) Schematic of the cross‐sectional anatomy of the upper arm in the imaged region (red dashed line in A). The approximate locations of the fields of view for the three nerves are outlined with grey rectangles. C) Schematic morphology of peripheral nerves. The nerve is organized in fascicles that contain the nerve fibers. The vascular supply is classified according to its location into epineurial, perineurial, and endoneurial vessels. D–F) Three representative ultrasound images of the three different nerves and their environments. The nerves are highlighted in purple. The areas shaded in green visualize the variance of the nerve locations in the whole dataset.

**Figure** **1** illustrates the data acquisition, the anatomy of the imaged region, the morphology of peripheral nerves, and representative US images of the three targeted nerves. Figure 1A shows the OPUS system used in this study, an iThera Medical Acuity Echo prototype, and the approximate region at the upper limb (red dashed line), a few centimeters proximal of the elbow, where OPUS images were acquired of the three major peripheral nerves in the arm: the ulnar, median, and radial nerve. Each nerve was imaged in two different locations on each arm of the 12 volunteers, resulting in a dataset of 135 images (45 ulnar, 47 median, 43 radial). A qualitative sketch of the cross‐sectional anatomy in the imaged region of the arm is given in Figure 1B, with grey rectangles outlining representative fields of view for the three nerves. Figure 1C shows the anatomy and vascular environment of peripheral nerves: nerve fibers are sheathed in lipid‐rich myelin and clustered in fascicles. The nerve's substructures are covered in collagen‐rich connective tissue: the epineurium wraps the nerve, the perineurium wraps the fascicles, and the endoneurium wraps single nerve fibers. Accordingly, the supplying blood vessels are classified by their location as epineurial, perineurial, and endoneurial vessels. Collectively, we refer to these vasa nervorum as intraneural vessels.

Figure 1D–F shows representative US images of the ulnar, median, and radial nerves, respectively, which are highlighted in purple. The depth of the imaged nerves’ centers was 7.10 ± 3.40 mm for the ulnar nerves, 8.93 ± 2.87 mm for the median nerves, and 14.93 ± 3.50 mm for the radial nerves. The green shaded areas in Figure 1D–F visualize the variance of the nerve locations in the dataset. These areas also serve as references for the spectral contrast of the nerve tissue. Within these regions, we can identify the typical anatomical environments of the three nerves. The ulnar nerve is superficially located next to the triceps with only smaller vessels (e.g., the ulnar collateral arteries) in its immediate vicinity. The median nerve is close to the brachial artery in a region with several large vessels (e.g., brachial and basilic vein). The radial nerve lies significantly deeper, usually between the brachialis and brachioradialis muscles.

Due to their different environments, the three nerves are not equally well suited for OPUS imaging. The ulnar nerve is the most accessible due to its superficial location and the absence of strong absorbers in its vicinity. The big vessels in the median nerve's vicinity dominate the contrast and can make it difficult to assess the nerve's OA contrast if, for example, the nerve is below a vessel or if imaging artifacts extend into the nerve region. In addition, the pulsation of an artery close to the median nerve might create motion artifacts that reduce image quality. The radial nerve lies deep in muscle tissue, which absorbs much light and leads to a very low OA contrast of the nerve and its immediate surrounding. These observations illustrate the fact that the penetration depth of optoacoustic imaging systems depends strongly on the imaged tissue and its absorption in the used wavelength range. Peripheral nerves at less than 1 cm depth (ulnar nerve) can be imaged with high data quality, while deeper nerves below muscle (radial nerve) have a reduced contrast due to light absorption.

To understand the amount of information that is available within the nerve segmentations, we investigated the cross‐sectional surface area of the nerves relative to the image resolution. The cross‐sectional surface area of the imaged nerves is 6.5 ± 4.1 mm^2^ for the ulnar nerves, 9.6 ± 4.4 mm^2^ for the median nerves, and 5.7 ± 3.0 mm^2^ for the radial nerves. With an image pixel's area of 0.01 mm^2^ (100 µm × 100 µm), a nerve contains on average 732 ± 422 pixels. Factoring in the system resolution of around 0.04 mm^2^, one arrives at a size ratio between nerve area and resolution area of approximately 183 ± 106.

### 2.2 Data‐Driven Spectral Unmixing Reveals Tissue‐Specific Optoacoustic Contrast

Raw MSOT imaging data are subject to spectral coloring due to wavelength dependent light absorption in tissue. Since reliable fluence correction is still an unsolved problem for clinical hand‐held systems, we embrace the fact that we work with raw (initial pressure) spectra. We performed a data‐driven spectral unmixing of the MSOT data in order to extract the tissue‐specific contrast provided by the multispectral images. In contrast to simple linear unmixing, a data‐driven unmixing method can capture spectral variations due to wavelength‐dependent fluence attenuation. In addition, our method of choice (regularized non‐negative matrix factorization (NMF), see Experimental section C.2) can be steered towards biologically reasonable spectra via suitable regularization, unlike other data‐driven methods like vertex component analysis (VCA). Our method explains the spectral MSOT data better than linear unmixing or VCA with a relative mean squared error of 0.72%, compared to 3.94% and 3.44% for linear unmixing and VCA, respectively.

**Figure 2:**
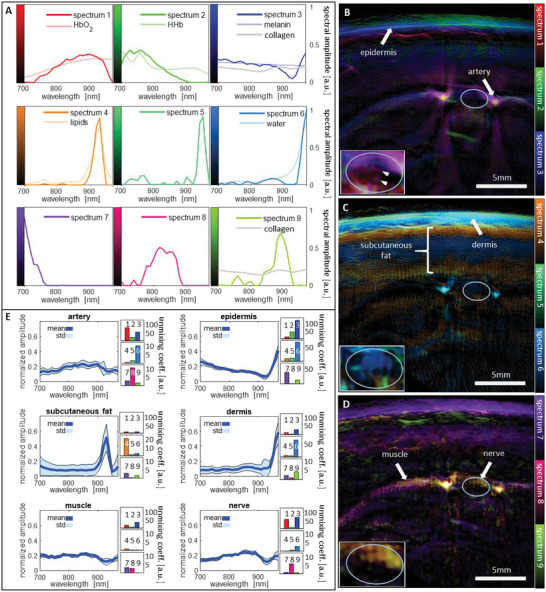
Spectral unmixing of the MSOT data. A) The spectral unmixing algorithm found nine distinct spectral components that agree with features of endogenous chromophores (hemoglobin, lipids, water, melanin, collagen). B–D) Spectral contrast of a representative ulnar nerve, visualized by color coding three components per image. The spectral data inside the nerve is shown with adjusted contrast and slightly smoothed in the lower left of each panel. E) Mean and standard deviations of the normalized spectra of the structures that are highlighted with white arrows in (C–E)—the artery to the right of the nerve, the epidermis, the subcutaneous fat, the dermis, the muscle, and the ulnar nerve. To the right of the graphs, the corresponding mean unmixing coefficients are displayed. HbO2: oxygenated hemoglobin, HHb: deoxygenated hemoglobin.

Since nerves are strongly vascularized and contain lipid‐rich myelin and collagen‐rich connective tissue (Figure 1C), their spectral OA contrast is expected to be a mixture of the absorption spectra of hemoglobin, water, lipid, and collagen. **Figure** **2** shows that the data‐driven spectral unmixing successfully captures the specific contrast of endogenous chromophores in the imaged tissues and, contrary to simple linear unmixing, can extract small spectral variations that are hidden behind the dominant absorptions of melanin, hemoglobin, lipids, and water.

The qualitative accuracy of the unmixing is validated in a representative image of an ulnar nerve. Figure 2A shows the nine fundamental spectral components that the unmixing algorithm extracted from the multispectral OA image data, i.e., every measured spectrum is represented as a weighted sum of these components. Four of these spectra closely resemble the absorption spectra of prominent endogenous chromophores: oxygenated and deoxygenated hemoglobin (spectra 1 and 2, respectively), lipids (spectrum 4), and water (spectrum 6). Spectrum 3 has the common slope of the spectra of melanin and collagen, but differs from both in the regions of lipid and water absorption. Instead, the weak absorption peak of collagen around 900 nm is captured in spectrum 9. Since the absorption by collagen will have the strongest effects in this wavelength region, spectrum 9 is expected to relate to collagen contrast. The remaining three spectral components (spectra 5, 7, and 8) capture variations of the spectra, presumably due to the light absorbed in superficial layers. While spectrum 5 can capture variations of blood contrast in the presence of lipids and water, being located in between and overlapping with the peaks of the respective spectra, spectrum 7 is able to represent the effects of absorption by the melanin in the skin by varying the contrast in the lower wavelength range. Spectrum 8 can adjust the contrast in the central wavelength range that captures the main blood contrast. Unlike these highly interpretable spectra, the spectral components identified by VCA do not properly resemble literature spectra and are additionally influenced by system noise (see Figure S1, Supporting Information).

Figure 2B–D visualizes the contrast of the nine spectral components in a representative image of an ulnar nerve and its environment by color‐coding three of the components in each image. The location of the nerve is indicated by a white ellipse. The inlays show an enlargement of the nerve with adjusted dynamic range to reveal the intraneural contrast that is otherwise hidden next to the strong signal of blood vessels. The spectral contrast in Figure 2B highlights the melanin in the skin line, the blood vessels in the skin, and two blood vessels to both sides of the nerve (presumably the ulnar collateral arteries). The strong optoacoustic contrast of these tissues is in agreement with the fact that blood and melanin are the strongest absorbers in tissue. Figure 2C shows strong contrast from spectra 4–6 in the superficial layers, highlighting the dermis and the subcutaneous fat. The fact that the subcutaneous fat extends until the depth of the nerve is not correctly reconstructed from the OA signal data due to filtering of the acquired signals to suppress low‐frequency noise and to achieve a better contrast of smaller structures, like nerves and vessels. The perfused tissues below—muscle and nerve—have good contrast in Figure 2D.

To qualitatively validate the accuracy of the unmixing results, Figure 2E shows the normalized spectra and standard deviations of six tissues that can be identified in the multispectral images together with their decomposition into the nine spectral unmixing components. The corresponding tissue segmentations are shown in Figure S2A (Supporting Information). The unmixing correctly captures the contrast of the oxygenated hemoglobin in the artery with spectrum 1, the contrast of water and melanin (epidermal melanocytes) in the epidermis with spectra 3 and 6, and the lipid contrast of the subcutaneous fat with spectrum 4. Since deoxyhemoglobin is present in healthy tissue only in mixtures dominated by oxyhemoglobin, and collagen has a low absorption in general, these two chromophores are difficult to unmix. The additional fact that the absorption spectra of these chromophores lack unique features in the considered wavelength range leads to ambiguities in the unmixing. As a consequence, spectra 2 and 3, which both have a slope similar to the spectra of deoxyhemoglobin, collagen, and melanin, can be used to explain spectral data related to any of these chromophores. Most notably, spectrum 3 is often present as a component of blood spectra, for example, in the spectrum of the artery. The strong contribution of spectrum 9 to the spectrum of the dermis indicates light absorption by dermal collagen. The spectra of nerve and muscle are similar to the spectrum of oxyhemoglobin due to the fact that both tissues are well perfused with blood. The ultrasound image (see Figure S2B, Supporting Information) shows that the optoacoustic contrast of the muscle does indeed stem from the absorption inside the muscle tissue and not from the fascia, that can be seen as a strongly reflecting layer above the muscle. The dip in the wavelength range of lipid absorption (900–940 nm) indicates the light attenuation due to the subcutaneous fat layer above these tissues.

The inlays in Figure 2B–D show the intraneural spectral contrast with two small absorbers (marked by arrows) in Figure 2B that indicate the presence of two small intraneural blood vessels. While there is a clear water contrast visible inside the nerve in Figure 2C, the expected lipid contrast of myelin is not visible. A possible reason for this is the attenuation of light in the respective wavelengths by the subcutaneous fat. The nerve has good contrast in the inlay in Figure 2D, showcasing the ability of the data‐driven unmixing to represent spectral data in deep tissue by capturing the spectral variations due to light attenuation, which are encoded in spectra 7–9.

### 2.3 OPUS Visualizes the Vasa Nervorum in Unprecedented Detail

**Figure 3:**
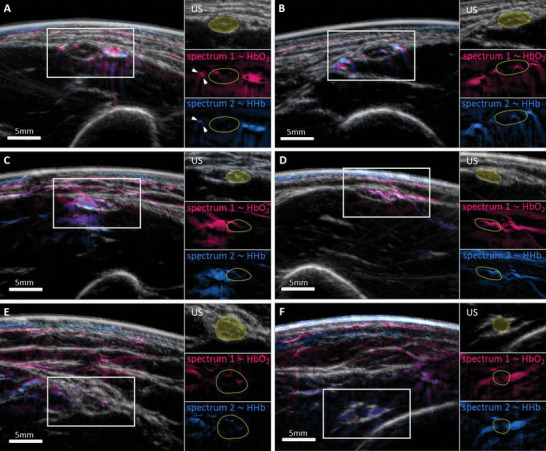
Visualizing the vasa nervorum. Overlay images of ultrasound (US, gray) and two spectral OA components associated with oxygenated (spectrum 1, red) and deoxygenated (spectrum 2, blue) hemoglobin. The single components in the regions outlined with rectangles are shown in three small panels next to the images with the nerve highlighted in yellow. A,B) Two ulnar nerves with intraneural vessels clearly visible in the OA channels. Arteries (presumably the ulnar collateral arteries) and veins at comparable depths can be spectrally distinguished. C,D) Two ulnar nerves with vessels branching into the nerve. E) A median nerve with visible intraneural vessels. F) A radial nerve with two accompanying vessels of medium size, and a small (presumably epineurial) vessel on top.

A direct application of the spectral unmixing is the visualization of the vasa nervorum which play a central role in several neurological disorders. To this end, **Figure** **3** shows that OPUS can visualize this vasculature with very good contrast, exceeding the capabilities of other in vivo nerve imaging modalities. All panels of Figure 3 show images of US contrast (gray) overlaid with two OA channels related to hemoglobin contrast (spectrum 1—oxyhemoglobin in red, spectrum 2—deoxyhemoglobin in blue) for six different peripheral nerves. The colormap has been changed relative to Figure 2 to show vessels with the intuitive red and blue contrast.

Figure 3A,B demonstrates the high MSOT contrast of blood vessels running along and inside of two superficial ulnar nerves. In Figure 3A, three blood vessels are clearly visible in the MSOT image to the right of the ulnar nerve. While the middle of these vessels, which is the ulnar collateral artery, could also be identified via its pulsation in the US images or via Doppler, the very small presumably epineurial vessels to the left of the nerve and the other intraneural vessels, which are clearly visible in the MSOT images (marked by arrows), cannot be visualized with US. Figure 3C,D shows two ulnar nerves with supplying vessels branching into the nerves visible in the MSOT channels, another detail of the vasa nervorum that cannot be visualized with US. Figure 3E,F shows images of a median and radial nerve, which showcase MSOT's ability to visualize vasculature around and within nerves that are deeper than 1.5 cm and below muscle tissue. The median nerve in Figure 3E with the brachial artery to its left has clearly visible intraneural vessels, while the radial nerve in Figure 3F is accompanied by two medium‐sized vessels with clear hemoglobin contrast even though the nerve is below a layer of muscle tissue. A small epineurial vessel is even visible on top of the nerve. Intraneural blood flow could previously only be visualized in patients with severe neuropathy,^[^ ^3^ ^]^ making the images in Figure 3 the first visualizations of intraneural vessels in healthy probands in vivo with a handheld system.

**Figure 4:**
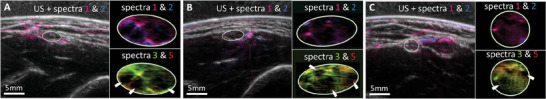
Visualizing the internal structure of nerves. The internal organization of nerves into fascicles and their vascular supply are visualized with good contrast by data‐driven spectral unmixing. Each panel shows an overlay of US and MSOT hemoglobin contrast (spectra 1 and 2) along with two enlarged images of a nerve with contrast from spectral components 1 and 2 (hemoglobin contrast) and components 3 and 5 (contrast related to collagen and hemoglobin). A,B) show two ulnar nerves, C) shows a median nerve.

The spectral contrast of arteries and veins at similar depths differs significantly, with arteries and veins having a stronger contrast in spectral components 1 and 2, respectively. This can clearly be seen, e.g., in Figure 3A, where the ulnar collateral artery that is accompanied by two veins could be identified via its pulsation (see also the brachial artery and vein in **Figure** **4**C, which can be distinguished via their compressibility). The fact that spectral components 1 and 2 are related to the contrasts of oxy‐ and deoxyhemoglobin shows that these differences result from differences in blood oxygenation. This qualitative distinction between arteries and veins at comparable depths is also demonstrated close to the system resolution limit of about 200 µm in panel A, where only one of the very small vessels (marked by arrows) is visible in spectral component 2, indicating that it is a vein.

The visualization of the vasa nervorum strongly depends on the depth and environment of the nerve, as shown by the percentages of nerves for which epineurial or other intraneural vessels could be visually identified (**Table** **1**). Vessels are identifiable in two thirds of the imaged ulnar nerves, less than half of the median nerves, and in only a few radial nerves. In addition, a diffuse blood contrast is visible in almost all of the 135 imaged nerves, indicating the contrast of subresolution vasculature, which is a valuable feature for the assessment of nerve perfusion.

**Table 1:** Visible vessels of the vasa nervorum. Numbers of nerves for which individual vessels could be visually identified in MSOT images

Visible vessels	Ulnar nerve (n = 45)	Median nerve (n = 47)	Radial nerve (n = 43)
Epineurial (pct.)	30 (67%)	17 (36%)	3 (7%)
Peri‐ or endoneurial (pct.)	16 (36%)	11 (23%)	1 (2%)

### 2.4 OPUS Can Access the Internal Structure of Peripheral Nerves

Figure 4 shows that OPUS can resolve the internal structure of peripheral nerves, i.e., the organization of nerve fibers into fascicles. The honeycomb‐like contrast, which can usually also be visualized with high‐frequency ultrasound, is visible in MSOT in spectral channels related to blood and collagen contrast. Thus, MSOT can link the structure of nervous tissue to its function.

The three panels of Figure 4 show overlay images of US and vascular contrast of two ulnar (A and B) and a median nerve (C), which are outlined with ellipses. The respective two subpanels display the intraneural contrast of spectra 1 and 2 (hemoglobin contrast) and spectra 3 and 5 (contrast associated with collagen and hemoglobin). The colormap for components 3 and 5 has been changed relative to Figure 2 to provide a better visual quality of the intraneural contrast. While the upper subpanels only show the intraneural vessels, fascicles can be clearly identified in the lower subpanels as negative contrast (marked with arrows) outlined by the contrast of the connective tissue and the intraneural vasculature. The images also again emphasize the intraneural vascular detail that is accessible with MSOT.

We could not consistently see the expected lipid contrast of the myelin sheaths as an increased signal of spectral component 4 (lipid peak) inside the fascicles. Due to light absorption in the subcutaneous fat, the spectra of all structures below this layer (artery, muscle, nerve) exhibit a dip in the lipid peak region (see Figure 2E), which suggests that spectral coloring is the reason for the missing fascicular lipid contrast.

### 2.5 Correlation Metrics Reveal Differences between the MSOT Contrast of Nervous and Reference Tissue

Going beyond representative images, we performed a correlation analysis on the whole dataset to confirm the observation that OPUS can capture the specific spectral contrast of peripheral nerve tissue. The goal was to understand how different spectral components mix in tissues, and which mixtures are observed in the data. We also sampled reference spectra from the nerves’ surroundings to investigate how the spectral contrast of nerves differs from that of other tissues at similar depths. In particular, we were interested to see, if we can find indicators for the lipid content of the myelin sheaths in the spectral data in the presence of spectral coloring due to subcutaneous fat.

We focus on the ulnar nerve in the rest of the paper because of the superior quality of the spectral data acquired in these more superficial nerves.

We investigated the spectral mixtures quantitatively using two correlation metrics—the Sørensen‐Dice coefficient (DSC) and the Pearson correlation coefficient (PCC) (see Experimental section C.4). In the context of spectral components, the DSC can be interpreted as the tendency of two components to mix: low DSCs indicate that components rarely mix, while high values indicate that components often appear together. The PCC in turn quantifies the type of mixtures: positive values for fixed ratio mixtures, low absolute values for random mixtures, or negative values for competitive mixtures.

**Figure 5:**
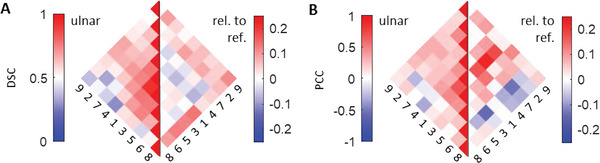
Correlation analysis of the spectral data acquired from ulnar nerves. A) Pairwise Sørensen‐Dice coefficients (DSC) of the spectral components of the ulnar nerve and their differences from the reference spectra that were sampled from the surrounding tissue. B) Pairwise Pearson correlation coefficients (PCC) of the spectral components of the ulnar nerve and their differences from the reference spectra that were sampled from the surrounding tissue. The spectral components are reordered for better visual interpretability.

**Figure** **5** shows the correlation metrics for the ulnar nerve and the reference data; the pairwise DSCs of the spectral components in Figure 5A and the pairwise PCCs in Figure 5B. The results for the median and radial nerves can be found in Figure S3 (Supporting Information). The left half of each panel displays the metrics for the spectra inside the nerve segmentations, while the right side shows the differences between correlations in nervous tissue and reference tissue sampled from the nerve's surrounding, wherein positive and negative values indicate higher and lower correlations inside nerve ROIs, respectively.

The results in Figure 5 confirm the biological accuracy of the unmixing results by reproducing the expected spectral mixtures in tissue and the expected differences between nervous and reference tissue. In particular, they show that in nervous tissue, relative to reference tissue, all components that relate to features of the collagen spectrum have higher mutual correlations, and that hemoglobin contrast is stronger correlated to lipid and collagen contrast. This observation is in agreement with the expected perfused connective tissue contrast within the nerve, and with the presence of the lipid‐rich myelin sheaths in the fascicles.

The left side of Figure 5A shows that spectra 1 and 3 have the highest DSC with each other, and also high DSC with all other spectra, confirming that they are the main components used for blood contrast, which is the dominant contrast of MSOT. Spectra 4 (lipid peak) and 8 have a very low DSC in general, confirming that spectrum 8 encodes blood contrast in deeper tissue below the subcutaneous fat, and highlighting the ability of data‐driven unmixing to capture such spectral coloring effects. The low DSC of spectra 2 and 6 (water peak) shows that spectrum 3 with its small peak at 970 nm encodes the water contrast in mixtures with blood instead of the pure water spectrum 6, demonstrating that the introduced metrics help to interpret unmixing results.

The left side of Figure 5B shows that the PCC values are generally similar to the DSC values (left side of Figure 2A), showing that spectral components that often mix do so in stable mixtures, while competitive components rarely mix. The differences between the PCCs of nervous and reference tissue show that spectrum 1 (oxyhemoglobin) has higher correlations with spectra 2‐5 and 7, which relate to blood, lipid, and collagen contrast, reproducing the expected contrast of perfused connective tissue and lipid‐rich fascicles in the nerve. In addition, spectrum 9 (collagen bump) correlates strongly with all decreasing spectra (collagen slope; spectra 2,3, and 7), what suggests a relation to collagen contrast. The negative correlations of spectra 6 with spectra 2 and 3 show that intraneural water contrast in mixture with blood is preferably encoded with spectrum 3.

Even though the tissue environments and the depths of the nerve and reference tissue are different for the three nerves, their Pearson correlations are very similar. The fact that we performed the Box‐Cox transformation independently for the three nerves and obtained a power parameter that varies monotonously with depth hints at a fluence adjusting effect of this transformation.

### 2.6 Hierarchical Clustering Finds Specific Details of Nervous Tissue in MSOT Data

**Figure 6:**
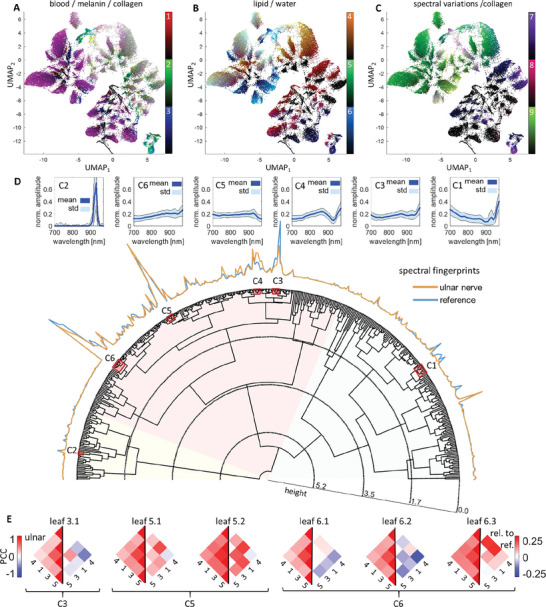
Hierarchical cluster analysis and UMAP embeddings of data acquired in ulnar nerves. A–C) UMAP embeddings of the standardized unmixing coefficients of the ulnar nerve dataset show the complexity of MSOT data. The three versions are colored as in Figure 2. D) Polar dendrogram of the hierarchical clustering tree. The spectral fingerprints, i.e., the relative number of pixels contained in each leaf, are plotted along the semicircle for the ulnar nerve data (orange) and the reference data (blue). Six clusters, labeled C1‐C6 are highlighted in red, with the mean and standard deviations of the spectral shape given in the graphs arranged above the spectral fingerprints. E) Pairwise PCC of the four spectral components present in all the six largest leafs of the clustering tree (called leaf 3.1, 5.1‐2, and 6.1‐3 according to the clusters they belong to).

**Figure** **6**A–C shows the full complexity of the MSOT data with a UMAP embedding of the standardized unmixing data (see Experimental section C.5) of the ulnar nervous and reference tissue in three versions colored as in Figure 2B–D. The UMAP algorithm arranges the data in a plane in which every point represents an OA spectrum in the dataset and seeks to keep the spatial and statistical relations between points as close to the original 9D data as possible. Every cluster in this visualization represents the data from one of the 512 = 2^9^ possible mixtures of the nine spectral unmixing components (Figure 2A). Due to this intricate structure of the data set, the correlations in Figure 5B potentially suffer from an averaging effect that cancels out variations in correlations within the subclusters of the data.

We performed a hierarchical clustering of the unmixing data (see Experimental section C.5), showing that mixtures of specific spectral unmixing components are consistently used to represent similar spectral contrast, allowing to target specific tissue contrast via clusters in the data. Analyzing the correlations in leafs of the clustering tree, we confirm that the correlations shown in Figure 5 indeed are affected by averaging effects and show that differences in correlations are specific to leafs of the clustering tree, i.e., specific to the tissue context.

Figure 6D shows a dendrogram of the clustering tree for the ulnar nerve data. The height of branchings in the tree indicates the dissimilarity of the mean spectral shapes in the two subclusters. We found 409 mixtures to be present in the dataset, with a subcluster of 141 mixtures (34.5% of all leafs) containing most of the contrast. To arrange the 409 mixtures meaningfully, the hierarchical clustering was guided by the mean spectral shape, i.e., the average normalized spectrum. The first two branchings of the tree divide the data coarsely into spectra dominated by fat contrast (yellow shading), spectra that belong to the main blood and perfused tissue contrast (red shading), and spectra of superficial layers with strong water contrast, like skin (green shading).

The relative numbers of pixels contained in each leaf of the clustering tree are plotted along the semicircle delineated by the leafs of the tree, both for the segmented nerve data (red line) and the sampled reference data (blue line). These plots are interpreted as spectral fingerprints of nervous tissue and reference tissue, respectively. Several regions display clear differences. Six interesting clusters, labeled C1–C6, are highlighted by red boxes in the clustering tree: the largest clusters (C3–C6), clusters with clear differences between the ulnar nerve and reference (C1, C3, C4), and specific contrasts (C1, C2; skin and lipid contrast). The six small graphs above the spectral fingerprints show the mean spectral shapes and standard deviations of the six clusters, demonstrating that mixtures are consistently used to represent similar spectral shapes. This implies that specific tissue contrasts can be targeted via hierarchical clustering of spectral OA data. For example, cluster C1 contains specific spectral mixtures of skin and is therefore not expected in nerve tissue, and cluster C2 contains the pure lipid contrast. Clusters C3–C6 represent the main MSOT contrast, containing spectral shapes similar to those of blood vessels, muscle tissue, and nervous tissue (see Figure 2E). We quantified the information content of the unmixing on the level of mixtures by computing the entropy of the spectral fingerprints of the combination of ulnar nerve and reference spectra. Our unmixing method achieved an entropy of 3.85, compared to 3.05 for VCA unmixing, which demonstrates that the sparsity prior on the coefficient vectors maximizes this quantity.

Figure 6E shows the PCCs of the four spectral components that are present in all of the six biggest leafs in the clustering tree (left sides, spectra 1, 3, 4, 5) and the difference between the PCCs of nervous an reference tissue (right sides). The PCC values in nervous tissue consistently agree with the results in Figure 5B. However, while leafs 5.1, 5.2, and 6.3 show similar correlation differences as in Figure 5B, the leafs 3.1, 6.1, and 6.2 show significant deviations in PCC. A variation of correlation differences is expected in different substructures. For example, while the perfused connective tissue of the nerve and the fascicles are unique to nervous tissue, blood vessels will have a similar spectral contrast within and outside of nerves. Additional details of the leafs in clusters C3–C6 are shown in Figure S4 (Supporting Information).

## 3 Discussion

With this pilot study on peripheral nerve imaging using a handheld OPUS system, we demonstrate that hybrid OA and US imaging can access multiple structural and functional parameters of healthy superficial peripheral nerves when combined with a suitable data processing pipeline. The OPUS images of the vasa nervorum (Figures 3 and 4) and the connective tissue of peripheral nerves (Figure 4) achieve a level of detail that has previously not been possible with a handheld OPUS system. Moreover, a dedicated analysis of the data revealed that spectral statistics carry detailed information about the specific spectral contrast of nervous tissue, thereby building a methodological foundation for OA radiomics in peripheral neurology and other medical fields. Since OPUS can be easily integrated into the clinical workflow, the study confirms that this modality has the potential to become a routine tool for the assessment of pathological changes in peripheral nerves.

Peripheral neuropathy, in particular diabetic neuropathy, affects millions of people and can cause serious complications and disabilities (e.g., amputation).^[^ ^5^, ^12^ ^]^ Screening for neuropathy, its most prevalent complication, currently does not employ any imaging method, but consists of physical examination and questionnaires, which implies that functional loss is already apparent when symptoms are detected. Despite being a worldwide health issue, the specific mechanisms underlying this polyneuropathy are poorly defined,^[^ ^31^ ^]^ though vascular and metabolic factors are thought to play pivotal roles.^[^ ^5^ ^]^ As demonstrated in this pilot study, OPUS can assess both vascular and metabolic features of peripheral nerves simultaneously in vivo.

The sensitivity of ultrasound Doppler is insufficient to detect blood flow in healthy nerves.^[^ ^3^ ^]^ Increased blood flow in sagittal Doppler imaging has been detected mostly in nerve pathologies and is therefore interpreted as nonphysiological.^[^ ^3^, ^32^ ^]^ The demonstrated high contrast of the vasa nervorum in MSOT images (see Figure 3) in healthy volunteers opens the possibility of investigating early pathological changes in the vascular supply of nerves. Indeed, ex vivo and animal studies provide diverse findings regarding the vasa nervorum of diabetic patients, including IV shunting, vascular occlusions, and endothelial hyperplasia.^[^ ^5^, ^7^ ^]^ Clinical surveys have also reported reduced perfusion of the diabetes‐affected nerve, a change in microcirculation, and endoneurial hypoxia,^[^ ^5^ ^]^ which correlate with neurophysiological outcome. Some of the abovementioned changes can be seen in very early stages of disease and precede functional loss and therefore clinical symptoms.^[^ ^5^ ^]^ Improved visualization of the neural vasculature, thus, could give new insights into early disease pathogenesis. In this regard, in our measurements of healthy and young individuals, intraneural vessels were visualized with good contrast, and veins and arteries could be distinctly identified. Therefore, arterio‐venous shunting and other pathological changes in nerve vasculature are likely visible in MSOT. In fact, MSOT‐based oxygen saturation estimation has already been used to detect vascular malformations.^[^ ^33^ ^]^

By accessing the water and lipid contrast in peripheral nerves, OPUS imaging can potentially identify several further changes related to diabetic neuropathy. The associated mild nerve enlargement, might be explained by swelling^[^ ^4^ ^]^ due to augmented amounts of osmotically active intracellular sorbitol,^[^ ^6^ ^]^ whose excessive metabolism is an important pathogenic mechanism of diabetic neuropathy.^[^ ^7^, ^34^ ^]^ Regarding lipid contrast, sural nerve preparations from patients with diabetes show alterations in myelin sheaths up to full segmental demyelination^[^ ^7^ ^]^; theoretically this would appear in MSOT as a decrease in OA contrast in the wavelength range of the lipid peak. Ultimately, OPUS could uncover potential abnormal changes that precede functional loss and hence serve as a screening tool and follow‐up examination on diabetic peripheral polyneuropathy.

With increasing sensitivity in diagnostics, OPUS might further elucidate or even uncover new aspects in pathological processes. It could potentially show abnormal changes that precede functional loss and hence serve as a screening tool and follow‐up examination on peripheral polyneuropathy. Understanding dysfunctional processes is crucial to find powerful solutions in prevention and treatment, while early therapy can change the course of most diseases profoundly.

As an imaging tool with additional contrasts that can be added to the broadly used ultrasound, OPUS could also help clinical examiners identify nerve tissue. For example, the detection of peripheral nerves or plexi with ultrasound for surgical or invasive treatments (e.g., regional anesthesia) is a common clinical procedure. Even without being the structure of interest, visualization is important to avoid damaging small nerves in the immediate vicinity during invasive procedures like placing a central venous catheter. However, in some cases, nervous tissue cannot be visualized with US because of a variation in anatomy, a small size, or poor contrast. Potential harm can arise due to poor visualization and a subsequent lack of identification.^[^ ^2^ ^]^ In this regard, combining the demonstrated ability to extract tissue‐specific features with machine learning methods for nerve detection and segmentation is a very promising approach.

While spectral unmixing of OA data has the potential to quantitatively determine chromophore concentrations in tissue, several factors limit the feasibility of this task. Spectral coloring due to light fluence attenuation, the similarity of absorption spectra of chromophores (e.g., melanin, collagen, and deoxyhemoglobin), and algorithmic biases can lead to biologically implausible unmixing results. In general, the question of whether a contribution of a spectral component truly indicates the presence of the corresponding chromophore is often not addressed in the literature with sufficient detail. A correlation analysis as shown in Figure 5 can reveal these implausibilities and thereby help to interpret the results, to optimize unmixing algorithms, and most importantly, to prevent incorrect conclusions being drawn from spectral unmixing results. In addition, our approach to unmix the raw (initial pressure) spectra data and capture spectral coloring effects in the unmixing components avoids the problems associated with the unreliability of current fluence correction methods for clinical optoacoustic handheld systems.

The introduced hierarchical clustering of the spectral data (Figure 6) shows that a dedicated data analysis pipeline is necessary to assess the details in spectral contrast. A simple probabilistic model for the unmixing coefficients allowed us to standardize the contrasts of different spectral components and analyze their correlations, even though their contributions to the overall contrast varied by orders of magnitude. The clustering in turn gives access to these correlations on the level of specific tissues. This rich source of information in clinical OPUS datasets is the ideal foundation for OPUS radiomics studies.

In this pilot study, we focused on healthy individuals and our findings suggest the possibility to detect morphological and metabolic changes in pathologic nerves. This conjecture needs to be investigated in further studies in patients with pathologically altered nerve anatomy or function. Moreover, the findings need to be correlated against existing tools including electrophysiology, Doppler US, and MRI. A crucial limitation for this application in neuroimaging is the low penetration depth of OA imaging. The best contrast by far was obtained for the very superficial ulnar nerve. While focal neuropathies in nerves that lie deeper, like the proximal ischiadic nerve, can currently not be accessed with OA imaging, further advances in the technology that improve image quality deeper in tissue are anticipated and will expand its applicability.

In summary, we demonstrated that OPUS is a versatile modality for peripheral nerve imaging. With its tissue‐specific label‐free optical contrast of endogenous chromophores, OPUS could improve nerve localization in difficult situations. Moreover, OPUS’ capacity to assess a nerve's lipid, water and collagen contrast and its vascular environment potentially expands the field of peripheral nerve imaging to a broad range of neuropathies by illuminating features of nerve morphology and function that have been hidden so far.

## 4 Experimental Section

### Imaging Systems

Two imaging systems were used in this study: a commercial US system for localization of the peripheral nerves and a hybrid OPUS system to acquire the coregistered OA and US data.

The OPUS system is a custom prototype of the Acuity Echo system (iThera Medical GmbH, Munich, Germany); it has a tunable laser that illuminates tissue with laser pulses of ≈8 ns duration with an energy of 16 mJ and a repetition rate of 25 Hz, thereby staying within the energy exposure limits defined by the American National Standards Institute. The acoustic part of the system consists of a circularly curved linear US transducer array with a 6 cm radius, 145° angular coverage, and 256 piezo elements, which were used both for reflection‐mode US imaging (≈5 MHz excitation frequency) and OA detection (4 MHz central detection frequency). The probe is filled with heavy water as a coupling medium. Acoustic signals are recorded at a sampling frequency of 40 MHz.

Hybrid MSOT and US imaging is realized with a schedule that acquires OA data at a rate of 25 Hz, cycling through 28 different wavelengths (700–970 nm in steps of 10 nm), while US data is acquired in between OA acquisitions at a rate of 6.25 Hz. The US system is operated in a synthetic aperture mode, acquiring reflection data from 256 single transducer transmission events with half the detector array receiving the response. The resulting data consist of multispectral data stacks that are acquired at ≈0.89 Hz with 4 US frames acquired per MSOT data stack.

The wavelength range was selected in the NIR‐I window to allow for optimal penetration of light into tissue, while the uniform sampling in that range was chosen to provide a good trade‐off between high information content and limited motion artifacts. This scheme was shown to provide high‐quality spectral data.^[^ ^19^, ^28^ ^]^

### Study Protocol

The three major distal nerves of the brachial plexus (ulnar, median, and radial) of 12 young healthy volunteers were investigated with OPUS. The three nerves were imaged at two different locations of the arm on both sides ( = 12 acquisitions per person, 144 total). Informed consent was obtained after the nature and possible consequences of the study were explained. The ethics committee of the Technical University of Munich reviewed the study and had no objections to the publication of the data. Basic personal parameters (age, sex, height, and weight) and data regarding body composition (BMI, body fat, arm diameter) were also collected. The latter parameters were recorded because the amount of body fat potentially changes the nerve tissue contrast due to the lipid‐rich myelin sheaths of nerve fibers. To avoid a distortion of the data by varying absorption in the skin, this study was conducted exclusively with light‐skinned volunteers. For examination, the volunteer lay on a bench facing the examiner. A summary of the study cohort is given in **Table** **2**.

**Table 2:** Study cohort. Basic personal and body composition parameters

n = 12 (8m, 4f)	Age [Year]	Height [m]	Weight [kg]	BMI [kg m^−2^]	Bodyfat [%]	Arm diameter [cm]
Mean ± std	29.1 ± 3.2	1.79 ± 0.11	71.8 ± 16.3	22.2 ± 2.9	16.0 ± 4.4	25.9 ± 2.7
Min ‐ max	24–36	1.60–1.93	49.0–107.0	17.6–28.7	7.0–25.0	21.0–30.0

For localizing the nerves, an experienced examiner (anesthetist) screened the arm with a commercial US device. The main peripheral nerves were identified and localized well with US, based on their typical appearance in US images with a reflecting outline and a honeycomb‐like contrast within. Anatomical landmarks along the progression of the nerve were used for confirmation. Once the respective nerve was identified, the region of interest was centered and the location marked. Snapshots of the ultrasound image at that specific site for a first qualitative segmentation of the nerve and to help re‐identify it on the Acuity Echo US images were taken.

After locating the nerve with the Acuity Echo US image, OA data were acquired for ≈10 s (≈10 multispectral frames). The raw US data were acquired for the last 10 recorded US frames. Because of the different features of the Acuity Echo US and the commercial system, a qualitative segmentation of the nerve in the Acuity US image by photograph was documented.

After cleaning the data (missing data, strong motion, or other artifacts), a total of 135 multispectral frames were available for further analysis. Quantitative segmentation of nerve tissue was performed in the reconstructed Acuity Echo US images by an expert using the qualitative segmentations obtained during the measurements for guidance.

### Data Analysis: Image Reconstruction

Both OA and US images were reconstructed in a 4 × 4 cm field of view at the center of the detector ring and at a resolution of 100 µm, using speed of sound values of 1397 m s^−1^ and 1465 m s^−1^ for the coupling medium in the detector cavity and the imaged tissue, respectively.

The US images were reconstructed from the acquired synthetic aperture data with a delay‐and‐sum algorithm after preprocessing. The data were bandpass filtered (3–6 MHz) and processed with a spiking deconvolution.^[^ ^35^ ^]^ For the delay‐and‐sum algorithm, the time‐of‐flight was determined and included the refraction of acoustic waves at the interface between the tissue and detector cavity to achieve a correct coregistration with the OA data. Finally, the delay‐and‐sum data was integrated via a mixed averaging and maximum intensity approach and logarithmically transformed to obtain the US images.

OA images were obtained from the acquired OA signal data after bandpass filtering (500 kHz–8 MHz), a deep‐learning‐based denoising of the sinograms,^[^ ^28^ ^]^ cropping the signals according to the field of view, model‐based reconstruction with refraction and impulse response correction^[^ ^26^, ^27^ ^]^ that was regularized with both a Tikhonov regularizer to filter out the noise due to the limited detector coverage, and a Laplacian regularizer to counteract subresolution artifacts. The regularization parameters were determined via the L‐curve.

### Data‐Driven Spectral Unmixing

To eliminate the influence of artifacts (acoustic reflections below bones, noise above the detector membrane, regions with bad coupling, etc.), regions of interest containing meaningful spectral data were manually segmented, resulting in a dataset of ≈12 M spectra. To extract the spectral contrast from this dataset, the spectra were blindly unmixed via a non‐negative matrix factorization (NMF)^[^ ^36^ ^]^ into nine spectral components, using mixed Frobenius and entrywise L^1^‐regularization. More precisely, arranging the spectra in a non‐negative matrix $S\in R^{N\times 28}$, *S* ≥ 0, where *N* is the number of spectra that are considered and the second dimension contains the values at the 28 different wavelengths, the following optimization problem was solved

(1) $$\begin{array}{ccc} \left(W,H\right): & = & \arg\min_{\left(W,H\right)\geq 0}\;\frac{1}{2}∥S-\mathrm{WH}{∥}_{F}^{2}+\lambda_{1}\left(∥W{∥}_{1}+∥H{∥}_{1}\right)\\ & & +\;\frac{1}{2}\lambda_{F}\left(∥W{∥}_{F}^{2}+∥H{∥}_{F}^{2}\right) \end{array}$$

where $∥M{∥}_{F}:={\left(\sum_{i,j}m_{i,j}^{2}\right)}^{1/2}$ and $∥M{∥}_{1}:=\sum_{i,j}\left|m_{i,j}\right|$ denote the Frobenius norm and the entrywise L^1^‐norm of a matrix *M* = (*m*_i,j_)_i,j_, respectively, and *M* ≥ 0 is an entrywise inequality, meaning that *M* is a non‐negative matix. The matrices $W\in R^{N\times 9}$ and $H\in R^{9\times 28}$ contain the coefficients and spectral components, respectively. The number of components and regularization parameters *λ*_1_ = 80 and *λ*_F_ = 20 were selected via parameter space exploration and meaningfulness of the resulting spectral components, yielding a relative error ${∥S-WH∥}_{F}^{2}/{∥S∥}_{F}^{2}$ of 0.72%. The strong entrywise L^1^‐regularization was chosen to promote a maximally sparse decomposition of the spectra, guided by the fact that the spectral contrast of biological tissue is composed by a small number of dominant chromophores. The unmixing was carried out using the scikit‐learn implementation of NMF.^[^ ^37^ ^]^

To validate the performance of the NMF on initial pressure spectra, two alternative unmixing methods were implemented: linear unmixing with a non‐negativity constraint for six literature spectra (oxy and deoxy hemoglobin, water, lipids, melanin, and collagen) and VCA with a non‐negativity constraint for nine components. The linear unmixing was performed with a non‐negative linear least squares solver,^[^ ^38^ ^]^ while the VCA was carried out using the original algorithm^[^ ^39^ ^]^ followed by a non‐linear least squares optimization^[^ ^38^ ^]^ to obtain the non‐negative coefficients.

In order to compare the spectral information in the segmented nerve regions to the tissue environment, reference spectra were sampled from the ROIs, assuming a bivariate normal distribution $N(\left(\mu_{\mathrm{lat}},\mu_{\mathrm{ax}}\right),\mathrm{diag}\left(\sigma_{\mathrm{lat}}^{2},\sigma_{\mathrm{ax}}^{2}\right))$, where µ_lat_, µ_ax_ and *σ*_lat_,*σ*_ax_ are the pixelwise lateral and axial means and standard deviations of the locations of the considered nerves. These reference spectra are sampled for each of the nerves independently to account for the differences in the spectral environment due to fluence at different depths and due to the specifics of the surrounding anatomy.

### Probabilistic Data Model

Based on the sparsity of the NMF, a simple probabilistic model is proposed for the NMF coefficients that describes whether each spectral component is present in a tissue or not. This aspect is realized by a probabilistic mixture model with two components for the two corresponding situations where the respective coefficient is either zero or non‐zero. In addition, as the distributions of the non‐zero parts are strongly skewed, Box‐Cox power transformations were performed to increase the comparability of different spectral components and the validity of metrics like the Pearson correlation coefficient. Since light fluence is thought to be a major contributor to skewness of the data, this transformation can also be seen as a partial fluence adjustment that improves the comparability of spectra at different depths.

In summary, the categorical parts of the mixture models is modeled for the nine spectral components via Bernoulli random variables $m_{j}∼\mathrm{Bernoulli}\left(p_{j}\right),\;j=1,\text{…},9,$ where *p_j_* is the probability that the corresponding coefficient is non‐zero. On the level of the mixture components, the zero component is a deterministic variable $C_{j}^{\left(0\right)}∼\mathrm{Det}\left(0\right)$, while the non‐zero component $C_{j}^{\left(1\right)}$ follows a distribution that is determined by the Box‐Cox transformation of the non‐zero coefficient data with power parameter *β*_*j*_, which is determined via a maximum likelihood approach.

In order to compare the coefficients of different spectral components in an unbiased way (strong OA signal does not necessarily imply high clinical relevance), a standardization of the mixture models was introduced. Since the Box‐Cox transformation results in distributions that are qualitatively similar to a normal distribution, the variables $C_{j}^{\left(1\right)}$ were studentized to obtain variables with zero mean and unit standard deviation ${\tilde{C}}_{j}^{\left(1\right)}≔\left(C_{j}^{\left(1\right)}-EC_{j}^{\left(1\right)}\right)/\sigma_{j}$, where *σ*_*j*_ denotes the standard deviation of $C_{j}^{\left(1\right)}$. To keep the relation between very low values of these variables ${\tilde{C}}_{j}^{\left(1\right)}$ and the zero values, the deterministic components were shifted to the value − 3 following the 3*σ* rule that states that for a normal distribution, 99.7% of the distribution lie within 3*σ*. So, the standardized deterministic components ${\tilde{C}}_{j}^{\left(0\right)}∼\mathrm{Det}\left(-3\right)$ are introduced, which are mixed with the components ${\tilde{C}}_{j}^{\left(1\right)}$ via the unchanged categorical variables *m_j_*.

### Correlation Metrics

To compare the different spectral components on both levels of the model—the categorical and the continuous parts—correlation metrics are calculated. To see additional differences between nerve tissue and surrounding tissue, this correlation analysis was performed for the nerve data and the sampled reference data.

As correlation metric for the categorical variables, the pairwise Sørensen‐Dice coefficient was chosen between the variables *m_j_*,

(2) $${\mathrm{DSC}}_{j,k}≔\;\frac{2m_{j}\cdot m_{k}}{{\left|m_{j}\right|}^{2}+{\left|m_{k}\right|}^{2}}\in \left[0,1\right],\;j,k=1,\text{…},\;9$$

where the *m_j_* are interpreted as vectors, and $v\cdot w≔\sum_{j}v_{j}w_{j}$ denotes the scalar product of two vectors. The Sørensen‐Dice coefficient describes the amount of co‐occurrences of two spectral components relative to the total occurrence of the two components. High values indicate that two spectral components usually appear together, while low values indicate that the spectral components rarely mix.

To study the pairwise correlation between the continuous variables ${\tilde{C}}_{j}^{\left(1\right)}$, the Pearson correlation coefficients are computed,

(3) $$\rho_{j,k}≔\;\frac{\mathrm{cov}\left({\tilde{C}}_{j}^{\left(1\right)},{\tilde{C}}_{k}^{\left(1\right)}\right)}{\sigma_{j}\sigma_{k}}=E\left[{\tilde{C}}_{j}^{\left(1\right)}{\tilde{C}}_{k}^{\left(1\right)}\right]\in \left[-1,1\right],\;j,k=1,\text{…},\;9$$

where cov(X,Y)≔E[(X−EX)(Y−EY)] is the covariance of two random variables *X* and *Y*, *σ*_*j*_ denotes the standard deviation of ${\tilde{C}}_{j}^{\left(1\right)}$ and the second equality follows from studentization. The Pearson correlation therefore describes how two spectral components mix when they mix: positive values for mixtures with a more or less fixed ratio, small values for random mixtures, and negative values for competitive mixtures.

### Dimensionality Reduction and Hierarchical Clustering

To visually access the full complexity of the spectral data set, and to investigate higher order correlations qualitatively, the 9D standardized spectral unmixing data were embedded into 2D space with the uniform manifold approximation and projection (UMAP) algorithm for dimensionality reduction.^[^ ^40^ ^]^

The binary structure of the probabilistic model leads to a hierarchical structure in a natural way. Two spectra are in the same class if they are composed of the same spectral components. As a metric to compare the similarity of these classes, using the Euclidean distance between the mean L^2^‐normalized spectra of the clusters is proposed, which is a metric for the similarity of the spectral shape. This approach links the class back to the original spectral data, making the clustering transparent and interpretable. For clustering linkage, Ward's linkage is used that minimizes in‐cluster variance. The agglomerative hierarchical clustering was carried out using the implementation in the Matlab function “linkage.”

For the collection of nine spectral components, this approach leads to at most 2^9^ = 512 leaves of the clustering tree. To compare tissues based on this hierarchical clustering, the distribution of the spectral data of ROIs over the found clusters was determined. This distribution is called the spectral fingerprint of a spectral dataset. In particular, this approach gives a means to spectrally compare nerve tissue to the surrounding tissue.

## Conflict of Interest

Vasilis Ntziachristos is an equity owner and consultant at iThera Medical GmbH.

## Author Contributions

D.J. and H.I. contributed equally to this work. Conceptualization: D.J., H.I., W.S., and F.H. Methodology: D.J., H.I., F.H., and C.D. Investigation: D.J., H.I., W.S., and F.H. Visualization: D.J. Funding acquisition: V.N., G.S., and N.N. Project administration: V.N. Supervision: D.J., W.S., N.N., G.S., and V.N. Writing—original draft: D.J. and H.I. Writing—review & editing: D.J., H.I., F.H., C.D., W.S., N.N., G.S., and V.N.

## Supporting information

Supporting Information

## Data Availability

The data that support the findings of this study are available on request from the corresponding author. The data are not publicly available due to privacy or ethical restrictions.
