# Supplementary material for: Spotlight on Nerves: Portable Multispectral Optoacoustic Imaging of Peripheral Nerve Vascularization and Morphology
Source: Adv Sci (Weinh). 2023 Apr 24;10(19):2301322. doi: 10.1002/advs.202301322 (PMC10323611; doi:10.1002/advs.202301322)
Supplement: Supplementary file 1 — Supporting Information [file ADVS-10-2301322-s001.pdf]

ADVANCED SCIENCE
Open Access

Supporting Information

for *Adv. Sci.*, DOI 10.1002/advs.202301322

Spotlight on Nerves: Portable Multispectral Optoacoustic Imaging of Peripheral Nerve Vascularization and Morphology

Dominik Jüstel, Hedwig Irl, Florian Hinterwimmer, Christoph Dehner, Walter Simson, Nassir Navab, Gerhard Schneider and Vasilis Ntziachristos*

Supplementary Materials

Supplementary Figures

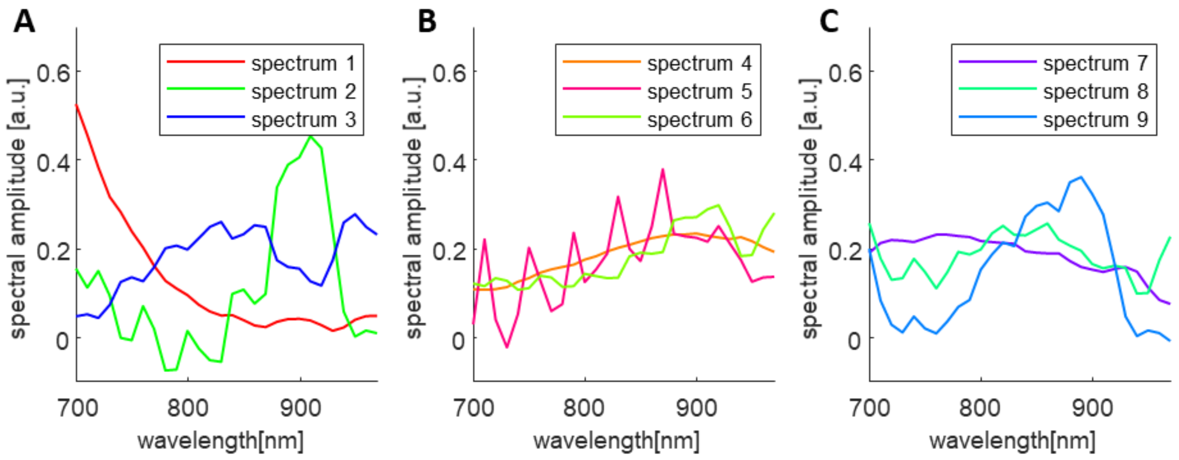

Supplementary Figure 1. Spectral components identified by VCA. The nine spectral components that were extracted from the spectral image data by vertex component analysis: (**A**) components 1-3, (**B**) components 4-6, (**C**) components 7-9.

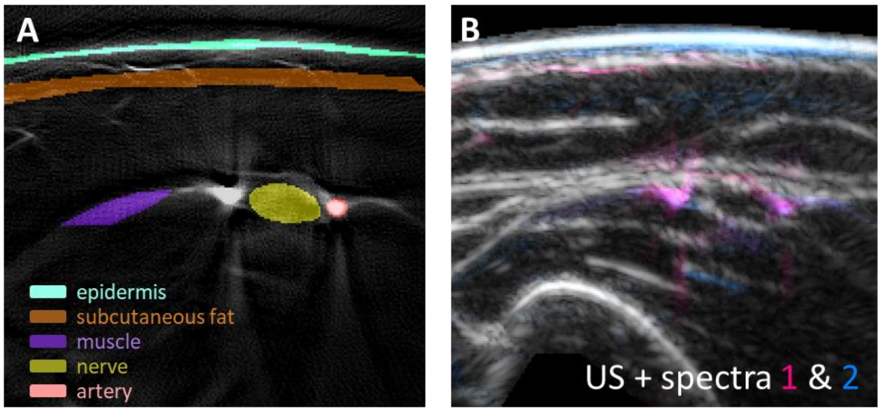

Supplementary Figure 2. Segmentation of tissues and ultrasound reference. (**A**) The segmentations of the six tissues analysed in Fig. 2. To properly capture the spectral contrast, the effect of fluence attenuation was reduced by only segmenting the superficial layers of bulk tissues, like subcutaneous fat or muscle. (**B**) The ultrasound image of the nerve shown in Fig. 2 with an overlay of spectral components 1 and 2. The fascia of the muscle can be clearly identified as a strong acoustic reflector in the ultrasound image.

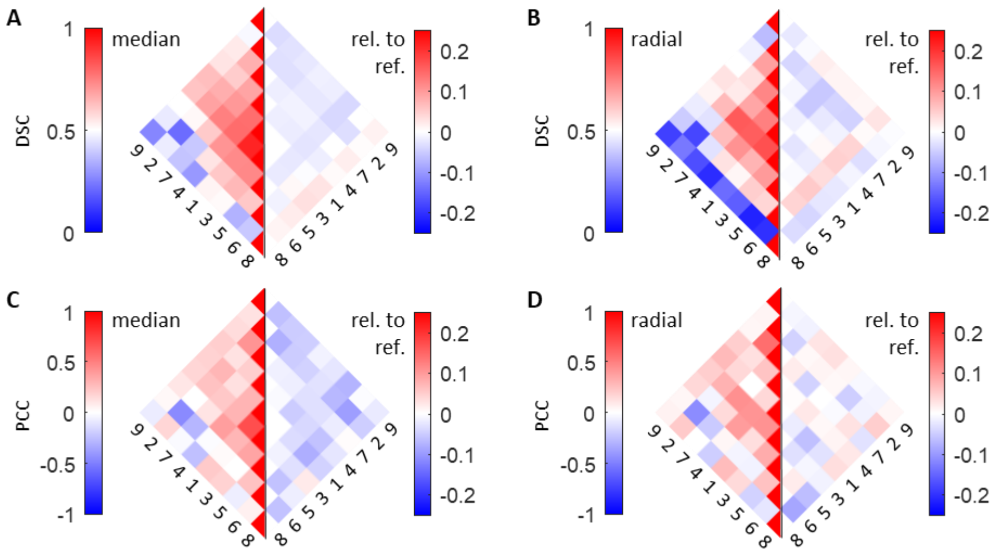

Supplementary Figure 3. Correlation analysis for the spectral data acquired in median and radial nerves. (**A**) and (**B**) Pairwise Sørensen-Dice coefficients (DSC) of the standardized spectral components of the median and radial nerve, respectively; and difference to the reference spectra that were sampled from the surrounding tissue. (**C**) and (**D**) Pairwise Pearson correlation coefficients (PCC) of the standardized continuous part of the spectral components of the median and radial nerve, respectively; and difference to the reference spectra that were sampled from the surrounding tissue.

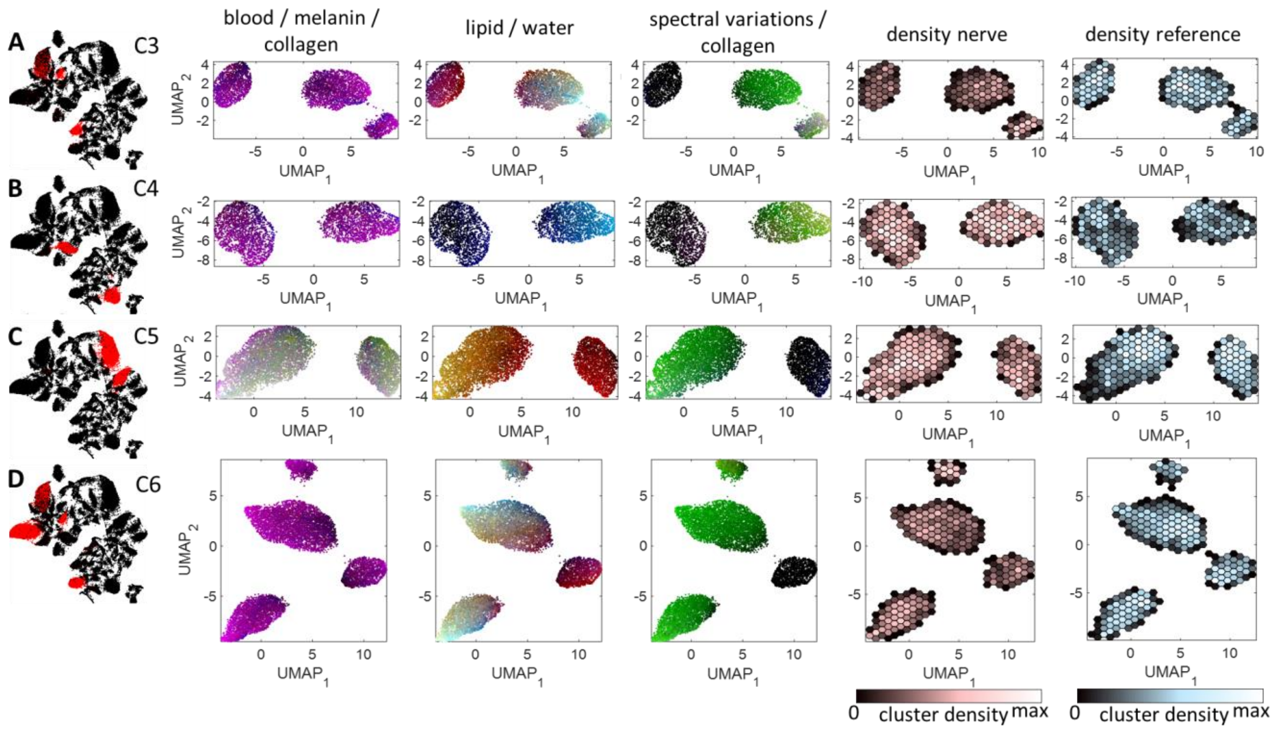

Supplementary Figure 4. Details of the four clusters C3-C6 highlighted in Fig. 6D. (**A**)-(**D**) The four clusters are localized in the full UMAP embedding (Fig. 6A-C) on the left. The UMAP embeddings of the clusters are color-coded as in Fig. 2 in the next three panels. Hexagonal density plots for the ulnar nerve data and the reference data are shown on the right.
